# *Hot topic:* Epidemiological and clinical aspects of highly pathogenic avian influenza H5N1 in dairy cattle

**DOI:** 10.3168/jdsc.2024-0650

**Published:** 2024-09-30

**Authors:** Zelmar Rodriguez, Catalina Picasso-Risso, Annette O'Connor, Pamela L. Ruegg

**Affiliations:** Department of Large Animal Clinical Sciences, College of Veterinary Medicine, Michigan State University, East Lansing, MI 48824

## Abstract

On March 24, 2024, the US Department of Agriculture (USDA) confirmed the first case of highly pathogenic avian influenza (HPAI) H5N1 clade 2.3.4.4b in dairy cattle. Following this initial detection, the virus was found to have spread to other species, including humans. Since then, at least 192 dairy herds across 13 states have tested positive for the virus. The emergence of HPAI H5N1 in dairy cattle poses a serious threat to the dairy industry, requiring an approach that integrates human, animal, and environmental health. Enhancing biosecurity, improving surveillance, and advancing research are critical to managing this challenge. Swift adaptation is needed to protect both public health and dairy production. Through this work, we discuss the epidemiological and clinical aspects of HPAI H5N1 in cattle by reviewing the spatiotemporal situation of the disease across the United States, the clinical signs and testing leading to a confirmed case, and the surveillance and control measures implemented at the national and state levels. Further, considering pathogenesis and risk factors, we discuss surveillance and control measures to implement within herds, and note areas where research is needed to understand how to control this emerging disease.

## Highlights

• The apparent cumulative incidence of HPAI H5N1 virus in dairy cattle has been increasing.
• There is an association between shedding load and clinical signs.
• Virus shed in milk is the most likely source of exposure.
• Practices that reduce exposure to milk from infected cows appear to be effective.
• The control strategy has focused on reducing contact rate and transmission.

## Graphical Abstract

**Figure:**
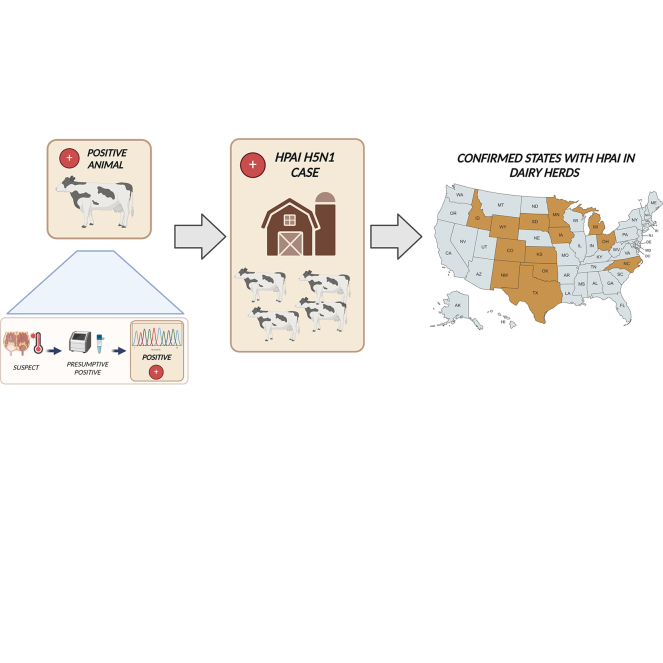
**Summary:** On March 24, 2024, the US Department of Agriculture confirmed the first case of highly pathogenic avian influenza (HPAI) H5N1 clade 2.3.4.4b in dairy cattle. Following this initial detection, the virus was found to have spread to other species, including humans. Since then, at least 192 dairy herds across 13 states have tested positive for the virus. Through this work, we discuss the epidemiological and clinical aspects of HPAI H5N1 in cattle by reviewing the spatiotemporal situation of the disease across the United States, the clinical signs and testing leading to a confirmed case, and the surveillance and control measures implemented at the national and state levels. Further, considering pathogenesis and risk factors, we discuss surveillance and control measures to implement within herds. We also note areas where research is needed to understand how to control this emerging disease. Graphical abstract created with BioRender.com.

On March 24, 2024, the USDA used molecular assays to confirm that unusual clinical signs that had been observed in dairy cattle in a Texas herd were caused by an infection of highly pathogenic avian influenza (**HPAI**) hemagglutinin type 5 and neuraminidase type 1 (**H5N1**) clade 2.3.4.4b (USDA-APHIS, 2024a). Genomic sequencing was used to determine that infection resulted from a spillover of the HPAI H5N1 virus from birds to cattle (Nguyen et al., 2024 [unpublished data]). The strain of HPAI H5N1 virus was the same strain that had been circulating in North America since at least December 2021 and was originally associated with severe disease and high mortality in wild birds (i.e., waterfowl, raptors, seabirds), commercial poultry facilities, and wild mammals (USGS, 2024). The spillover from birds to dairy cattle was unprecedented and raised concerns about cross-species transmission. Soon after infected dairy cattle were identified, infections were also detected in domestic cats, commercial poultry, and several species of small mammals (Burrough et al., 2024). In April, the Centers for Disease Control and Prevention confirmed that a dairy farm worker from one of the infected Texas herd had conjunctivitis caused by HPAI type A H5N1 infection, which represented the first transmission of the virus from cattle and humans (Uyeki et al., 2024).

The original Texas dairy herds that were eventually confirmed to be infected with HPAI H5N1 were initially identified based on observation of vague clinical signs affecting 10% to 15% of lactating cows (Oguzie et al., 2024). Reported clinical signs included respiratory and gastrointestinal disease, dramatically decreased milk production and abnormal milk with thick colostrum-like appearance (Oguzie et al., 2024). Additional clinical signs include increased SCC, reduced milk yield, fever, dehydration, nasal discharge, lethargy, and tacky or loose feces (Caserta et al., 2024). The changes in milk consistency and reduced milk yield are usually the most notable clinical signs of HPAI H5N1, likely due to recognition during milking.

Based on a survey of dairy farms confirmed to have infected cows (USDA-APHIS, 2024b), >80% (n = 12) of producers reported abnormal appearance of milk and decreased feed consumption. Among those reporting abnormal milk, 92% noted thickened milk and clots, and 70% mentioned yellow discoloration of milk. Producers estimated that reduced milk yield persisted for 8 to 15 d, abnormal appearing milk persisted for 3 to 6 d, and other clinical signs persisted for 6 to 17 d. Anecdotal reports of late-term abortions linked to infection are still being investigated. Analysis of sensor data from a single infected herd (personal information, ZR) showed a 5-fold increase in body temperature and drop in rumination alerts during the early stages of infection, rising from 5% to 35% daily. The same closed herd experienced a 22% drop in milk yield at the outbreak's peak, with normal production levels taking over 2 mo to recover. Bulk tank SCC more than doubled and took about 2 mo to return to pre-infection levels after the onset of clinical signs. While there is much uncertainty about the course of the disease, combined mortality and culling directly related to HPAI have been reported to account for 2% or less of the herd, but most of these cows were culled based on clinical signs (USDA-APHIS, 2024b). Causality between HPAI and death has not been confirmed. According to interviews by authors of this work with producers and veterinarians in Michigan, deaths directly related to HPAI are rare, with cases typically defined only by clinical signs and the presence of comorbidities (e.g., mastitis) not fully assessed, suggesting a good prognosis for most infected cows.

Clinical signs observed on infected farms are consistent with those reported in a recent experimental challenge (Baker et al., 2024 [unpublished data]) in which 2 nonpregnant lactating primiparous Holstein cows in late lactation received intramammary inoculation in 2 quarters with 1 mL of 1 × 10^5^ H5N1 clade 2.3.4.4b genotype B3.13. After inoculation, both cows developed mastitis and had positive California mastitis test results and abnormal milk (thickened, change in color, and presence of clots) from 2 to 14 d postinoculation (**DPI**). Rumination time also declined in the first 7 DPI. In the first 23 DPI, cow 1 dropped 71% and cow 2 dropped 77% compared with pre-exposure rumination time. Cows showed lethargy, reduced feed intake, self-resolving diarrhea, and nasal discharge. Although the progression of symptoms is not well defined, the observed clinical signs are consistent. We hypothesize that fever may decrease water intake, which in turn reduces milk yield and increases SCC due to concentration. The rise in SCC is likely also due to the migration of additional neutrophils into the mammary gland as part of a nonspecific immune response.

A herd-level diagnosis of HPAI H5N1 is initially based on observation of typical clinical signs followed by confirmation using a validated diagnostic test performed at an authorized laboratory. Herds are initially considered as “suspect” based on the presence of typical clinical signs or the combination of clinical signs and the presence of influenza A antigen in milk based on a USDA-approved and commercially available test kit (USDA-APHIS, 2024c). Preferred samples depend on lactation status of the suspect animals and are either milk (from lactating cows) or nasal swabs (from nonlactating animals), which must be submitted to an approved National Animal Health Laboratory Network (**NAHLN**; USDA-APHIS, 2024c) for identification of H5N1 RNA using real-time PCR. While the analytical sensitivity of PCR has not been formally estimated in cattle, its high sensitivity and specificity observed in other species supports its use in cattle as well (Spackman, 2020). When at least one sample from an individual cow in a herd tests positive, the herd is considered to be presumptively positive for the disease but infection status must be confirmed by samples that are submitted to USDA NVSL to identify HPAI H5N1 clade 2.3.4.4b using molecular assays or genome sequencing (USDA-APHIS, 2024c). A positive test result in a herd, based on testing from NVSL is, only then, called a confirmed positive case. Defining and identifying herd-level cases enables state officials to make decisions about movement restrictions to prevent further spread and to increase testing and surveillance for early detection of incident farms. Thus, for regulatory purposes cases are defined at herd-level based on at least one cow that has confirmed as positive for HPAI H5N1 clade 2.3.4.4b by testing at a USDA NVSL. We will use this regulatory definition when further referring to cases.

Observation of clinical signs is a sensitive screening tool for initial detection of infected cows and is used to identify cows for further laboratory testing (i.e., PCR). However, a negative association between cycle threshold (**Ct**) values of PCR tests in milk for H5N1 (i.e., viral shedding load) and clinical signs has been observed (Baker et al., 2024 [unpublished data]). This, together with the fact that (1) infected asymptomatic cows can still shed and spread the virus across the herd, (2) while infected, some cows can be in the latent phase in which they do not manifest clinical signs or shed the virus, and (3) lack of milk samples, the most sensitive samples, for testing nonlactating categories, indicates the need to further study diagnostic strategies and within-herd transmission dynamics. An option could be the use of antibody-detection methods (e.g., ELISA) to effectively identify infected animals while understanding the role of natural immunity on disease manifestations and shedding patterns is essential. However, the use of ELISA for HPAI H5N1 diagnosis in cattle is not yet validated nationally. Furthermore, understanding the dynamics of within-herd transmission is necessary to identify different diagnostic strategies and to enable producers to implement control measures such as segregation, culling, enhanced biosecurity, and increased monitoring of susceptible cows.

At present, it is difficult to fully determine the scope of infection with HPAI H5N1 in US dairy herds because most states have not required mandatory testing. Although the USDA is responsible for control of foreign animal diseases (such as foot and mouth disease) and can require mandatory test and removal policies for those diseases, HPAI H5N1 is not considered a foreign animal disease, and control programs are the responsibility of individual states. Individual states vary in their regulatory approaches to this disease and, to date, only Colorado has initiated mandatory testing of bulk tank milk.

As of August 23, 2024, based on testing performed by USDA 192 dairy herds in 13 states have been confirmed as infected with HPAI H5N1. However, these data reflect apparent rather than true incidence because many producers are unwilling to report suspected clinical signs in their herds, and thus incidence is likely underestimated. With the exception of a positive test in a backyard herd in Idaho, all cases have been confirmed in commercial dairy herds. After the initial herd in Texas was confirmed as infected, there was a steady increase in newly confirmed positive herds until the end of April after which cases sharply increased (Figure 1). Based on publicly available data from APHIS-USDA (2024), most farms with confirmed HPAI H5N1 infection have been located in Colorado (64 farms, 33%), Idaho (31 farms, 16%), Michigan (27 farms, 14%), and Texas (24 farms, 12%), whereas the remaining 9 affected states accounted for 25% of the cases. These top 4 affected states account for almost one-fourth (22.3%) of the US milk production (USDA-ERS, 2023). However, of the 10 US states that produce the most milk, infected herds have not been confirmed in California (at the time of article writing and acceptance, California had no confirmed cases of HPAI; 3 herds have since tested positive), Wisconsin, New York, Pennsylvania, or Washington. When considering the number of licensed dairy farms by state (Michigan approximately 980 herds, Texas 380, Idaho 450, and Colorado 110) the apparent within state herd-level incidence is 2.7%, 6.3%, 6.9%, and 58% for Michigan, Texas, Idaho, and Colorado, respectively. Apparent incidence in Colorado is notably higher than the other states. Although the reasons driving the incidence and weekly cases growth rate by state are still unclear, multiple factors, such as the number of farms per state, average herd size, cattle movement among herds within the state, clustering of herds, and the roles of private and governmental organizations are some of the factors that are expected to substantially influence disease spread and the willingness to conduct testing.

**Figure 1:**
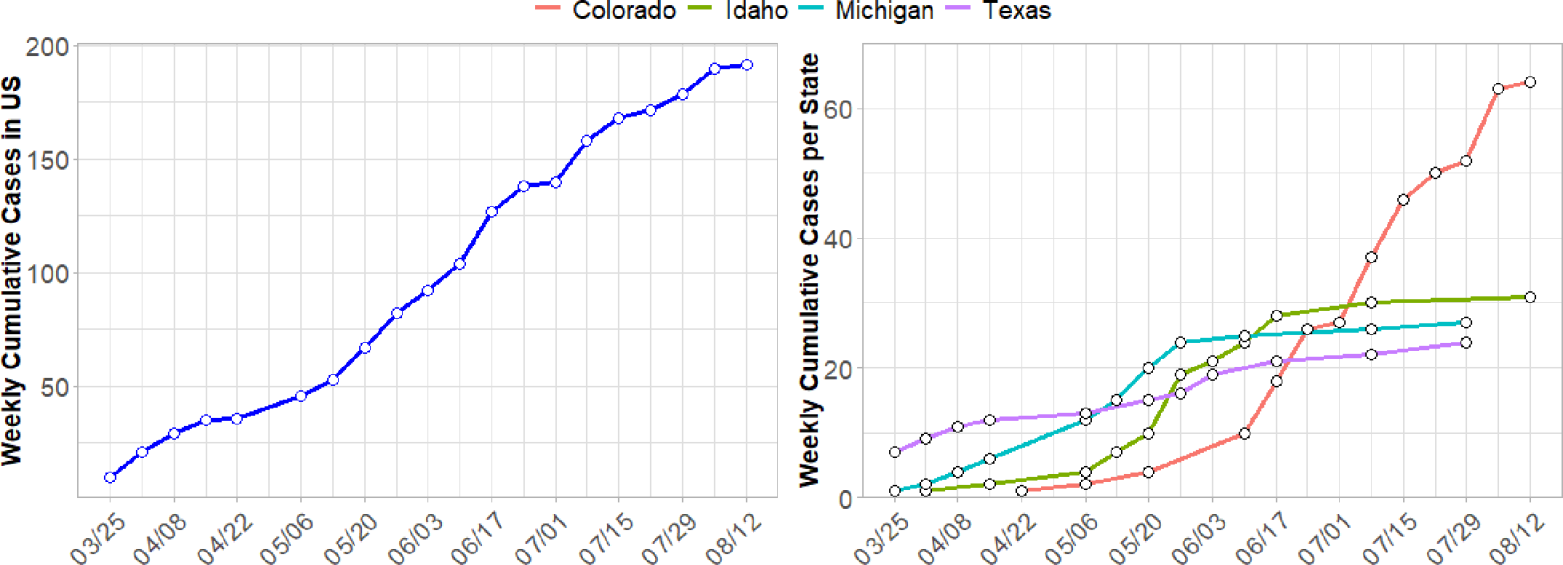
Cumulative confirmed cases (positive herds) of HPAI H5N1 reported per week in dairy herds in the United States (left), and highly affected states (Colorado, Idaho, Michigan, Texas; right) from March 25 to August 13, 2024.

Nationally, from the first case on March 25, 2024, to August 13, 2024, the number of confirmed infected herds increased by an average of 18.6% each week compared with the previous week. However, trends in cumulative confirmed herds have differed among affected states. Since its first outbreak on April 22, 2024, Colorado's cases have increased by 47.9% weekly. Similarly, Idaho and Michigan have shown steady weekly increases of 46.0% and 44.0%, respectively, indicating a consistent rise in cases over time. In contrast, Texas had a slower increase of 13.3% per week (Figure 1). It is important recognize that weekly changes in detection of infected herds cannot be interpreted as a direct measure of transmission, as detection of case-herds is confounded by differential spread dynamics, potential unwillingness of farmers to report, differences in density of dairy farms among regions, and proximity of neighboring farms in the states. It is very likely that the number of affected herds is underestimated. Underestimation of case herds is supported by a recent study that reported the presence of HPAI H5N1 in grade A pasteurized retail milk products in 17 states (Spackman et al., 2024).

Two important aspects of the epidemiology of HPAI H5N1 remain undefined: (1) how herds initially become infected, and (2) dynamics of transmission within a herd after initial infection. According to the USDA-APHIS (2024b) survey of 15 infected dairy herds and 8 poultry premises in Michigan, 3 potential transmission pathways were identified. The primary route was the introduction of infected animals into naïve herds, as seen in the first Michigan outbreak where asymptomatic infected animals were shipped from Texas. The survey also found that 6 of the 15 positive farms had introduced new cattle within 30 d before HPAI H5N1 was confirmed. Transmission through fomites, such as vehicles and people, is also a suspected risk factor. Results from Michigan showed that the majority (14/15) of the farms use the same coop for milk collection, and 8 shared the same deadstock removal service. Six dairy farms reported sharing workers with other farms, and workers from 5 farms shared housing, representing a potential source of interaction. Finally, as HPAI H5N1 genotype B3.13 was prevalent in wildlife and peri-domestic species in 5 of the affected dairies, suggesting a potential transmission route. Although these risk factors are intriguing, the survey data were a case series from infected farms and did not include data from unaffected farms; thus, risk factors cannot be quantified. Controlled studies are needed to identify and quantify the risk of these and other potential transmission pathways among dairy herds. Therefore, animal movement, visitors, shared vehicles, workers, and wildlife remain potential risk factors, but none have been confirmed.

Within a herd, the route of transmission among animals remains unknown. However, the viral load is extremely high in unpasteurized milk samples (Baker et al., 2024 [unpublished data]; Burrough et al., 2024). These findings suggest that contact with infected unpasteurized milk is a likely source of transmission. Thus, risk factors may include contaminated milking units, and milking-related procedures, such as the use of contaminated gloves during teat handling or poor teat disinfection. If milk is the major source of virus, then control practices need to be oriented to identify and segregate clinical and asymptomatic shedders while disinfecting milking equipment to limit the spread among cows.

Additional routes of within-herd transmission have been considered. Experimental aerosol inoculation of 4 Holstein heifer calves of 1 yr of age with 2 mL of 1 × 10^6^ HPAI H5N1 virus resulted in viral RNA detection in oropharyngeal swabs, ocular swabs, and saliva in one calf across multiple days but sporadically and not on consecutive days in the other 3 calves (Baker et al., 2024 [unpublished data]). The only clinical sign observed in these calves was transient nasal discharge. These data suggest that HPAI is present in the nares, and therefore airborne transmission might occur, but it is not likely to be a primary route of spread. Reinforcing evidence comes from an experimental challenge in ferrets with the bovine H5N1 virus, which reported that transmission through respiratory droplets was inefficient (Eisfeld et al., 2024). However, as most milk production occurs in housing systems where cows are in close proximity, air transmission could still play a meaningful role in the introduction and maintenance of the disease that still need to be further quantified. As airborne transmission has neither been confirmed nor ruled out, factors such as stocking density, segregation, and quarantine of newly introduced animals should be considered when developing prevention and control strategies.

Putative risk factors for infection with HPAI H5N1in dairy cows include age and stage of lactation. According to the USDA-APHIS (2024b) survey, the percentage of animals that exhibited clinical signs was 4% for first lactation, 7% for second lactation, and 9% for third or greater lactation. Additionally, 5% of dry cows exhibited clinical signs, whereas clinical signs were not detected in preweaning and nonlactating heifers.

In general, control of infectious diseases is based on 3 fundamental pillars: (1) reduction in contact rate and probability of transmission, (2) immunization of susceptible individuals, and (3) target interventions for infected animals. These measures are typically applied at both the herd and individual animal levels. As movement among herds is federally regulated, herd-level control has focused on reducing contact between infected and susceptible herds. In April 2024, the USDA issued a federal order to limit interstate movements of infected dairy cattle, requiring a negative test for HPAI H5N1 virus for lactating cows at an approved NAHLN laboratory before they can be moved across state lines (USDA-APHIS, 2024d). Within-states, regulatory requirements have varied. Some state governments have mandated testing protocols and enhanced biosecurity practices. Colorado has initiated weekly bulk-tank milk sampling from all dairy farms (CDPHE, 2024). Michigan's HPAI Risk Reduction Response Order, issued in May 2024, focuses on biosecurity measures for dairy herds and poultry flocks, including a designated biosecurity manager, secure perimeters, vehicle and individual disinfection, and maintenance of a visitor log (MDARD, 2024). Similarly, Iowa issued an order in June 2024 focused on surveillance, which requires testing dairy herds within a 20-km radius around infected poultry farms. Some states such as California and Florida, that have no reported cases, have restricted dairy cattle movement from affected states.

The USDA has launched a voluntary H5N1 Dairy Herd Status Pilot Program as an alternative screening tool to facilitate testing and identification of newly infected herds. This measure focuses on surveillance to monitor apparent incidence, prevalence, and distribution of HPAI H5N1 within the US dairy herd, aiding authorities in deciding how to reduce disease transmission rates. As of August 23, 2024, 26 herds in 9 states (including several states without any confirmed positive herds) have enrolled.

Immunization is a potentially important control procedure and the USDA has issued a “request for information” from companies capable of developing and producing vaccines for cows against H5N1 (USDA, 2024). However, due to international trade regulations, the adoption of vaccines as a control strategy remains uncertain (Cohen, 2024).

Based on identified putative risk factors and presumed viral shedding, producers can implement general infectious disease control measures on dairy farms. Some measures are standard practice, whereas others may require additional support. Maintaining a closed herd prevents the introduction of susceptible individuals, reducing transmission risk. Alternatively, acquiring animals from herds with negative test results and testing incoming animals individually can help. Positive or symptomatic animals should be segregated and milked last. Proper hygiene, such as teat disinfection and liner sanitization, is crucial. Newborns should not receive colostrum from infected dams, and all colostrum and milk should be pasteurized before feeding to calves. If economically viable, infected animals that exhibit typical clinical signs or are positive using diagnostic based tests should be isolated or implement early dry-offs to reduce the chance of transmission to susceptible animals within the herd by limiting effective contacts and reducing the shedding period. Quarantine is an option, but its effectiveness needs further assessment as intermittent shedding by previously infected cows is not well understood.

To develop effective control strategies, a clearer understanding of the multifactorial mode and magnitude of disease transmission and associated risk factors is needed. Information on the impact of the disease on productivity is also needed for estimating herd-level costs and guiding resource allocation. A major concern is the confirmation of 13 human cases related to HPAI H5N1 to date (CDC, 2024), which highlights the need to understand cattle-to-human and potential human-to-human transmission risks. Some of these questions are being addressed through ongoing experimental and field research projects, which is remarkable given the short time since the first case was reported. This rapid progress has been made possible through close collaboration between agencies, universities, and stakeholders. However, while much has been learned about the disease, there is still much to understand.

The emergence of HPAI H5N1 in dairy cattle represents a major and unprecedented threat to the dairy industry. The virus's ability to cross species underscores the urgent need for a One Health approach, recognizing the interconnectedness of human, animal, and environmental health. As the dairy industry confronts this new challenge, it is critical to mitigate the risks associated with HPAI in cattle by enhancing biosecurity measures, advancing research to better understand the disease, and developing robust surveillance systems. The industry must adapt swiftly to this emerging threat to ensure sustainable production and safeguard public health.

## References

[bib1] APHIS-USDA (2024). HPAI confirmed cases in livestock | Animal and Plant Health Inspection Service. https://www.aphis.usda.gov/livestock-poultry-disease/avian/avian-influenza/hpai-detections/hpai-confirmed-cases-livestock.

[bib2] Baker A.L., Arruda B., Palmer M.V., Boggiatto P., Sarlo Davila K., Buckley A., Ciacci Zanella G., Snyder C.A., Anderson T.K., Hutter C., Nguyen T.-Q., Markin A., Lantz K., Posey E.A., Torchetti M.K., Robbe-Austerman S., Magstadt D.R., Gorden P.J. (2024). Experimental reproduction of viral replication and disease in dairy calves and lactating cows inoculated with highly pathogenic avian influenza H5N1 clade 2.3.4.4b. PREPRINT. bioRxiv.

[bib3] Burrough E.R., Magstadt D.R., Petersen B., Timmermans S.J., Gauger P.C., Zhang J., Siepker C., Mainenti M., Li G., Thompson A.C., Gorden P.J., Plummer P.J., Main R. (2024). Highly pathogenic avian influenza A(H5N1) clade 2.3.4.4b virus infection in domestic dairy cattle and cats, United States, 2024. Emerg. Infect. Dis..

[bib4] Caserta L.C., Frye E.A., Butt S.L., Laverack M., Nooruzzaman M., Covaleda L.M., Thompson A.C., Koscielny M.P., Cronk B., Johnson A., Kleinhenz K., Edwards E.E., Gomez G., Hitchener G., Martins M., Kapczynski D.R., Suarez D.L., Alexander Morris E.R., Hensley T., Beeby J.S., Lejeune M., Swinford A.K., Elvinger F., Dimitrov K.M., Diel D.G. (2024). Spillover of highly pathogenic avian influenza H5N1 virus to dairy cattle. Nature.

[bib5] CDC (2024). CDC confirms three human cases of H5 bird flu among Colorado poultry workers. https://www.cdc.gov/media/releases/2024/s0725-three-human-cases-of-h5-bird-flu.html.

[bib6] CDPHE (2024). Colorado state veterinarian now requiring HPAI testing of commercial dairy cow operations | Colorado Department of Agriculture. https://ag.colorado.gov/press-release/colorado-state-veterinarian-now-requiring-hpai-testing-of-commercial-dairy-cow.

[bib7] Cohen J. (2024). Companies start work on bird flu vaccines for cows—Despite major hurdles. https://www.science.org/content/article/companies-start-work-bird-flu-vaccines-cows-despite-major-hurdles.

[bib8] Eisfeld A.J., Biswas A., Guan L., Gu C., Maemura T., Trifkovic S., Wang T., Babujee L., Dahn R., Halfmann P.J., Barnhardt T., Neumann G., Suzuki Y., Thompson A., Swinford A.K., Dimitrov K.M., Poulsen K., Kawaoka Y. (2024). Pathogenicity and transmissibility of bovine H5N1 influenza virus. Nature.

[bib9] MDARD (2024). Determination of extraordinary emergency HPAI risk reduction & response. https://www.michigan.gov/mdard/-/media/Project/Websites/mdard/documents/media/HPAI-Risk-Reduction-Response-Order.pdf?rev=d7a8d5bba77341c0bdfb4ae05c2836ed&hash=C258D640360EFF6882EF677A6513EA4C.

[bib10] Nguyen T.-Q., Hutter C., Markin A., Thomas M., Lantz K., Killian M.L., Janzen G.M., Vijendran S., Wagle S., Inderski B., Magstadt D.R., Li G., Diel D.G., Frye E.A., Dimitrov K.M., Swinford A.K., Thompson A.C., Snevik K.R., Suarez D.L., Spackman E., Lakin S.M., Ahola S.C., Johnson K.R., Baker A.L., Robbe-Austerman S., Torchetti M.K., Anderson T.K. (2024). Emergence and interstate spread of highly pathogenic avian influenza A(H5N1) in dairy cattle. PREPRINT. bioRxiv.

[bib11] Oguzie J.U., Marushchak L.V., Shittu I., Lednicky J.A., Miller A.L., Hao H., Nelson M.I., Gray G.C. (2024). Avian influenza A(H5N1) virus among dairy cattle, Texas, USA. Emerg. Infect. Dis..

[bib12] Spackman E., Spackman E. (2020). Animal Influenza Virus.

[bib13] Spackman E., Jones D.R., McCoig A.M., Colonius T.J., Goraichuk I.V., Suarez D.L. (2024). Characterization of highly pathogenic avian influenza virus in retail dairy products in the US. J. Virol..

[bib14] USDA (2024). Notification of U.S. Department of Agriculture's (USDA) request for information for highly pathogenic avian influenza (HPAI) vaccines for use in cattle. https://www.aphis.usda.gov/sites/default/files/notice24-09.pdf.

[bib15] USDA-APHIS (2024). Detections of highly pathogenic avian influenza (HPAI) in livestock. https://www.aphis.usda.gov/livestock-poultry-disease/avian/avian-influenza/hpai-detections/livestock.

[bib16] USDA-APHIS (2024). https://www.aphis.usda.gov/sites/default/files/hpai-dairy-national-epi-brief.pdf.

[bib17] USDA-APHIS (2024). Case definition–Avian influenza. https://www.aphis.usda.gov/sites/default/files/hpai-livestock-case-definition.pdf.

[bib18] USDA-APHIS (2024). Federal order requiring testing for and reporting of highly pathogenic avian influenza (HPAI) in livestock. https://www.aphis.usda.gov/sites/default/files/dairy-federal-order.pdf.

[bib19] USDA-ERS (2023). Milk cost of production estimates | USDA-Economic Research Service. https://www.ers.usda.gov/data-products/milk-cost-of-production-estimates/.

[bib20] USGS (2024). Distribution of highly pathogenic avian influenza H5 and H5N1 in North America, 2021/2022 | US Geological Survey. https://www.usgs.gov/centers/nwhc/science/distribution-highly-pathogenic-avian-influenza-north-america-20212022#overview.

[bib21] Uyeki T.M., Milton S., Abdul Hamid C., Reinoso Webb C., Presley S.M., Shetty V., Rollo S.N., Martinez D.L., Rai S., Gonzales E.R., Kniss K.L., Jang Y., Frederick J.C., De La Cruz J.A., Liddell J., Di H., Kirby M.K., Barnes J.R., Davis C.T. (2024). Highly pathogenic avian influenza A (H5N1) virus infection in a dairy farm worker. N. Engl. J. Med..

